# Exploring Effects of *C. elegans* Protective Natural Microbiota on Host Physiology

**DOI:** 10.3389/fcimb.2022.775728

**Published:** 2022-02-14

**Authors:** Kohar Annie B. Kissoyan, Lena Peters, Christoph Giez, Jan Michels, Barbara Pees, Inga K. Hamerich, Hinrich Schulenburg, Katja Dierking

**Affiliations:** Department of Evolutionary Ecology and Genetics, Zoological Institute, University of Kiel, Kiel, Germany

**Keywords:** *C. elegans*, microbiota, *Pseudomonas*, *Bacillus thuringiensis*, fertility, lifespan, colonization, pore forming toxin (PFT)

## Abstract

The *Caenorhabditis elegans* natural microbiota was described only recently. Thus, our understanding of its effects on nematode physiology is still in its infancy. We previously showed that the *C. elegans* natural microbiota isolates *Pseudomonas lurida* MYb11 and *P. fluorescens* MYb115 protect the worm against pathogens such as *Bacillus thuringiensis* (Bt). However, the overall effects of the protective microbiota on worm physiology are incompletely understood. Here, we investigated how MYb11 and MYb115 affect *C. elegans* lifespan, fertility, and intestinal colonization. We further studied the capacity of MYb11 and MYb115 to protect the worm against purified Bt toxins. We show that while MYb115 and MYb11 affect reproductive timing and increase early reproduction only MYb11 reduces worm lifespan. Moreover, MYb11 aggravates killing upon toxin exposure. We conclude that MYb11 has a pathogenic potential in some contexts. This work thus highlights that certain *C. elegans* microbiota members can be beneficial and costly to the host in a context-dependent manner, blurring the line between good and bad.

## Introduction

All plants and animals coevolved with their resident microbes, the microbiota, with which they form an entity, the metaorganism (Bosch and McFall-Ngai, 2011). In the metaorganism, the microbiota profoundly affects host health (Ezcurra, 2018; Haller, 2018), influencing disease prognosis (Wang et al., 2017) under different host health conditions (e.g., dysbiosis, pathogenesis, and immunosuppression) (Brown and Clarke, 2017). Microbiota-mediated protection is achieved when the microbiota mitigates or eliminates the impact of a pathogen on the host (Chiu et al., 2017). However, in contrast to the beneficial role of the microbiota in supporting host health, microbiota can have pathogenic potential in some contexts (reviewed in Stevens et al., 2021). In addition, the protective impact of some microbes can be costly for the host. For example, the bacterial endosymbiont *Hamiltonella defensa* protects aphids from parasitoids but decreases their lifespan (Vorburger and Gouskov, 2011). Thus, certain protective microbes can have divergent impacts on the host. Knowledge of these divergent effects of microbiota on the host is crucial, particularly for the development of microbiota-based therapeutic strategies (e.g., probiotics, prebiotics, and fecal microbiota transplantation), as adverse effects can be devastating, especially for an impaired host (Jochum and Stecher, 2020; Stevens et al., 2021).

The recent characterization of the *C. elegans* natural microbiota opened up the possibility to exploit the benefits of this simple yet powerful model system in host-microbiota research (Berg et al., 2016a; Dirksen et al., 2016; Samuel et al., 2016; Zhang et al., 2017). The *C. elegans* natural microbiota positively impacts various aspects of host physiology such as lifespan (Haçariz et al., 2021), development (Dirksen et al., 2020), body size (Zhang et al., 2021), and healthspan (Sonowal et al., 2017). Moreover, specific microbiota isolates protect *C. elegans* against pathogens or stressors (Montalvo-Katz et al., 2013; Berg et al., 2016b; Dirksen et al., 2016; Samuel et al., 2016; Kissoyan et al., 2019). However, *C. elegans* microbiota has also been shown to mediate trade-offs such as the microbiota-mediated increase in host developmental rate but a decrease in host resistance to heat stress (Slowinski et al., 2020), or an increase in pathogen resistance but a decrease in nematode fecundity (Montalvo-Katz et al., 2013). Also, the protective *C. elegans* microbiota member *Enterobacter* species accelerates development and enhances resistance to the pathogen *Enterococcus faecalis*, but decreases lifespan in the absence of the pathogen and becomes pathogenic in mutant *C. elegans* strains with disrupted Transforming Growth Factor (TGF)β/Bone Morphogenetic Protein (BMP) signaling (i.e., immunocompromised worms) (Berg et al., 2019). We are only just beginning to understand such microbiota-mediated diverging effects that reveal complex and multifaceted *C. elegans*-microbiota interactions (Zimmermann et al., 2019; Radeke and Herman, 2021).

Previously, we identified *C. elegans* microbiota isolates that efficiently protect the worm against pathogen infection (Kissoyan et al., 2019). The protective microbiota isolate *Pseudomonas lurida* MYb11 produces massetolide E, an antimicrobial cyclic lipopeptide of the viscosin group, which directly inhibits the growth of the Gram-positive nematicidal pathogen *Bacillus thuringiensis* (Bt). Another protective microbiota isolate, *P. fluorescens* MYb115, does not produce massetolide E and does not directly inhibit pathogen growth and may thus mediate protection through indirect, host-dependent mechanisms (Kissoyan et al., 2019). The extent to which MYb11 and MYb115 affect the host in diverging ways in different contexts has not been previously investigated. Here, we investigated the effects of the protective MYb11 and MYb115 on *C. elegans* lifespan, fertility, and intestinal colonization in the absence of a pathogen. We also assessed the ability of MYb11 and MYb115 to protect *C. elegans* against purified Bt toxins.

## Materials and Methods

### Worm Strains and Maintenance

The wild-type strain N2 (Bristol) was obtained from the Caenorhabditis Genetics Center (CGC, Minnesota, USA), the transgenic strain BJ49 *kcls6* [IFB-2::CFP] was obtained from the Leube Lab (RWTH Aachen University, Aachen, Germany). All worms were grown and maintained on nematode growth medium (NGM) seeded with the *Escherichia coli* strain OP50 at 20°C, according to the routine maintenance protocol (Stiernagle, 2006). Worm populations were synchronized and incubated at 20°C.

### Bacterial Strains and Maintenance

Natural *C. elegans* microbiota isolates *Pseudomonas lurida* MYb11 and *Pseudomonas fluorescens* MYb115 were used (Dirksen et al., 2016). The microbiota isolates and the *E. coli* OP50 control were grown on Tryptic Soy Agar (TSA) plates at 25°C and liquid bacterial cultures were grown in Tryptic Soy Broth (TSB) in a shaking-incubator overnight at 28°C. Peptone free medium (PFM, 3% agar) plates were inoculated with 750 µL OD_600nm_ of 10, of the overnight cultures and used to maintain worms from L1 to the L4 stage.

*Bacillus thuringiensis* (MYBt679 and MYBt407) spore aliquots were obtained following a previously established protocol (Borgonie et al., 1995) with minor modifications (Kissoyan et al., 2019). The Bt spore aliquots were prepared in bulk and stored at -20°C, with concentrations of 10^9^-10^10^ particles/mL for Bt679 and 10^3^-10^4^ particles/mL for Bt407. New Bt aliquots were freshly thawed for each assay.

### Fluorescent Bacterial Tagging

To fluorescently label the *C. elegans* natural microbiota isolates MYb11 and MYb115, we used a previously published protocol (Wiles et al., 2018) with minor modifications. We performed the bacterial mating overnight and used selective and differential media, MacConkey, to distinguish between *E. coli* and *Pseudomonas* spp. colonies to ensure the selection of the desired colonies. We further performed a confirmatory 16S rRNA PCR using primers SHP160(27f) (5’-GAGAGTTTGATCCTGGCTCAG-3’) and SHP161(1495r) (5’CTACGGCTACCTTGTTACGA-3’) and Sanger sequencing, in addition to targeted PCR(TN7) using primers WP11 (5’-CACGCCCCTCTTTAATACGA-3’) and WP256 (5’-CAGCTGCTCTCCTACTACGT-3’).

### *B. thuringiensis* MYBt679 (Bt679) Toxin Purification

Bt679 toxins were provided by Elena A. Andreeva and Dr. Guillaume Tetreau from the group of Dr. Jacques-Philippe Colletier (Univ. Grenoble Alpes, CNRS, CEA, Institut de Biologie Structurale, Grenoble, France). Bt679 toxins were purified and the required quality control was performed according to a previously published protocol (Zárate-Potes et al., 2020).

### Population Growth Assay

Population growth assays were done to measure the nematode fitness through extrapolating the offspring produced by a single worm after two generations according to a standardized protocol (Dirksen et al., 2016; Kissoyan et al., 2019). Here we used three L4 larvae that grew up from L1 to L4 stage on OP50, MYb11 or MYb115, respectively. Worms were picked onto assay plates containing a lawn of adjusted bacteria (OD_600nm_ of 10) mixed with Bt679 spores. Worm population size was measured after an incubation period of 5 days at 20°C. The population growth assay was performed at least three times in independent runs.

### Survival Assay

*B. thuringiensis* survival assays were done according to the standardized protocol (Nakad et al., 2016; Kissoyan et al., 2019; Zárate-Potes et al., 2021). Worms were grown on 6 cm PFM plates with lawns containing 75 µl of MYb11, MYb115 or OP50 (OD_600nm_ of 10) mixed individually with different concentrations of the Bt679 spores (1:5, 1:25, 1:50) or with the Bt407 spores (1:5). The pre-prepared Bt spore aliquots were stored in 1 mL tubes, at -20°C, with a concentration range of 10^9^-10^10^ particles/mL for Bt679, and 10^3^-10^4^ particles/mL for Bt407. These were freshly thawed immediately prior to every assay. Bt407 was used as a non-pathogenic Bt control (Sheppard et al., 2013; Zárate-Potes et al., 2020). Worm survival was assessed 24 hours post infection (hpi) as alive or dead using gentle touch with a sterile platinum wire. Similarly, for the survival assay with purified Bt679 toxin, MYb11, MYb115 or OP50 (OD_600nm_ of 10) were mixed individually with different dilutions (1:10, 1:50, 1:100, 1:1000) of the purified toxin or no toxin (0). Bt survival assays were done in three independent runs, each with four biological replicates per treatment group and around 30 worms per biological replicate.

### Lifespan

For the lifespan assay, eggs were harvested by bleaching and allowed to grow to L1 in M9 buffer overnight at 20°C. L1 larvae were transferred to PFM plates with lawns containing 750 µL (OD_600nm_ of 10) of OP50, MYb11 or MYb115 and raised at 20°C until they reached the L4 larval stage. We did not observe any developmental delay in any of the microbiota treatment conditions and the L4 stage was reached by 46 h in all three bacterial treatment conditions. L4 worms were then picked with a platinum wire pick to fresh PFM plates of the respective bacterium. All PFM assay plates were inoculated with 150 µL of the adjusted bacterial cultures in advance and stored in the fridge at 4°C. 30 min before the picking, plates were transferred to 20°C to ensure that the plates reached room temperature. Every 24 h, alive and dead worms were counted, and all living worms were picked to a new PFM plate until the worms stopped laying eggs. Worms were considered dead when they failed to respond to gentle touch with the platinum wire pick.

### Fertility

To measure nematode fertility in response to the different bacteria, worms were prepared as for the lifespan assay. However, single worms were picked to new plates daily, and the hatched offspring were counted until the worms reached the end of their reproduction period.

### Bacterial Colonization Assay

To assess differences in bacterial colonization (i.e. number of live bacteria present inside the *C. elegans* intestine after several washings), synchronized L4 larvae were transferred to new PFM plates every 24 h until they reached: L4, 24 h post L4 (Day 1), 72 h post L4 (Day 3) and 120 h post L4 (Day 5) stage. Worms were washed off the plates with M9T (M9+0.025% Triton-X100) and washed 5 times before being paralyzed with 10 mM tetramisole. This was followed by soft-bleaching, as described in (Dirksen et al., 2020), and the total worm number was counted and homogenized as described in (Pees et al., 2021). Homogenized worms were diluted (1:10 for L4, day 1 and day 3; 1:100 for day 5) and plated onto TSA plates, as well as the undiluted supernatant as a washing control. After 48 h at 25°C, colonies were counted, and the colony-forming units (CFUs) per worm were calculated. The soft bleaching treatment sterilized the worm surface sufficiently as the supernatant was almost always clear of viable bacterial cells.

### Confocal Laser Scanning Microscopy

To visualize the microbiota bacteria in the *C. elegans* intestine, the transgenic worm strain BJ49 was synchronized, and L1 larvae were exposed for 72 hours to fluorescently labeled bacteria (OP50::dTomato, MYb11::dTomato or MYb115::dTomato). Subsequently, young adult worms were washed with M9T to remove all bacteria that were outside the worms, and then these worms were mounted alive in 10 mM tetramisole on agar-padded object slides using high-performance coverslips (Carl Zeiss Microscopy GmbH, Jena, Germany). All visualizations were performed with a ZEISS LSM 880 confocal laser scanning microscope system (Carl Zeiss Microscopy GmbH). A 40× C-Apochromat water immersion objective with a numerical aperture of 1.2 (Carl Zeiss Microscopy GmbH) was applied, and Immersol™ W (2010) with a refractive index of 1.334 (Carl Zeiss Microscopy GmbH) was used as the immersion medium. The dTomato fluorescence of the bacteria and the CFP fluorescence of the worm intestinal structures were sequentially excited with 561 nm and 458 nm laser light, respectively, and detected with an Airyscan detector operated in the R-S mode (sensitivity mode) without using any emission filters. The system was controlled by the software ZEISS Efficient Navigation 2 (Carl Zeiss Microscopy GmbH). The processing of the Airyscan raw data was performed with the automatic Airyscan processing function of this software.

### Statistical Analyses

Statistical analyses were performed with RStudio (Version 1.4.1717). The following statistical tests were used: ANOVA and GLM analysis with Tukey multiple comparison tests (Hothorn et al., 2008) and False Discovery Rate (FDR) or Bonferroni (Benjamini and Hochberg, 1995) correction were used for the survival and bacterial colonization assays respectively. The lifespan data were analyzed with the Kaplan-Meier log rank test with FDR correction for multiple testing (Kaplan and Meier, 1958). Wilcoxon-Rank sum test with FDR correction for multiple testing was used for the bacterial colonization and population growth assays. The fertility data was analyzed using Kruskal-Wallis test, with Wilcoxon pairwise comparison and FDR correction. Statistical analyses are found in the **Supplementary Table**. Graphs were plotted using ggplot2 (Wickham, 2016) in R (Version 4.1.0) (R Core Team, 2021) and were edited in Inkscape (Version 1.0.1). All treatments were randomized using random codes on plates to prevent experimenter bias.

## Results

### MYb11 and MYb115 Affect Reproductive Timing, and MYb11 Affects Fertility and Lifespan

**Figure 1:**
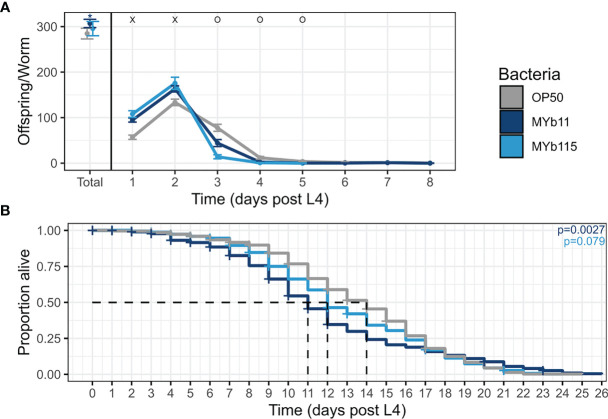
MYb11 and MYb115 affect reproductive timing, and MYb11 affects fertility and lifespan. **(A)** Daily brood size (hatched offspring) of *C. elegans* exposed to the two natural microbiota isolates, *P. lurida* MYb11 and *P. fluorescens* MYb115, compared to worms exposed to the food bacterium *E. coli* OP50. Hatched offspring were counted every day until the worms did not produce any more offspring for two consecutive days. Presented here are pooled data combined from 5 independent experiments (n= 75 (OP50), n=69 (MYb11), n=43(MYb115), also see **Supplementary Table**). “x” indicates significantly higher hatched offspring for worms on the natural microbiota isolates, while “o” indicates significantly higher numbers for worms on OP50. *p < 0.05, as determined by Kruskal-Wallis test, with Wilcoxon pairwise comparison and FDR correction. **(B)** Lifespan analysis of *C*. *elegans* N2 under different bacterial conditions on PFM plates. Alive, dead, and missing worms were counted until all worms were dead, i.e., failed to respond to the light touch of a platinum wire picker. Shown are pooled data combined from four independent experiments (n=573 (OP50), n=459 (MYb11), n=554 (MYb115), also see **Supplementary Table**). *P-*values were determined with Kaplan-Meier analysis and log-rank test, and are considered significant according to: *p < 0.05. Horizontal ticks represent censored data (missing worms).

As a first step to investigate the effects of the protective microbiota isolates *P. lurida* MYb11 and *P. fluorescens* MYb115 on *C. elegans* life-history traits, we grew worms on each of the microbiota isolates MYb11 and MYb115, or the control food bacterium *E. coli* OP50 and measured nematode fertility (i.e., hatched larvae). We found that both microbiota isolates affected reproductive timing and increased early reproduction up to 22% and 31% on MYb11 and MYb115 respectively, at day two of adulthood compared to worms on OP50 (**Figure 1A** and **Supplementary Table**). Moreover, worms on MYb11 showed a marginal, but statistically significant, increase in overall fertility.

We next asked if the protective microbiota impact *C. elegans* lifespan, e.g., due to a potential trade-off between the protective effect and lifespan. When combining the lifespan data from all runs, we found that the overall lifespan of worms was significantly reduced on MYb11 but not on MYb115 compared to worms on OP50 (**Figure 1B**). At the same time, it is important to note that the effect of MYb11 on lifespan was characterized by high variability, with a highly significant negative effect on lifespan in two independent experimental runs, but no significant effect in two other runs (**Supplementary Table**).

### MYb11 and MYb115 Colonize the *C. elegans* Intestine Throughout Adulthood

**Figure 2:**
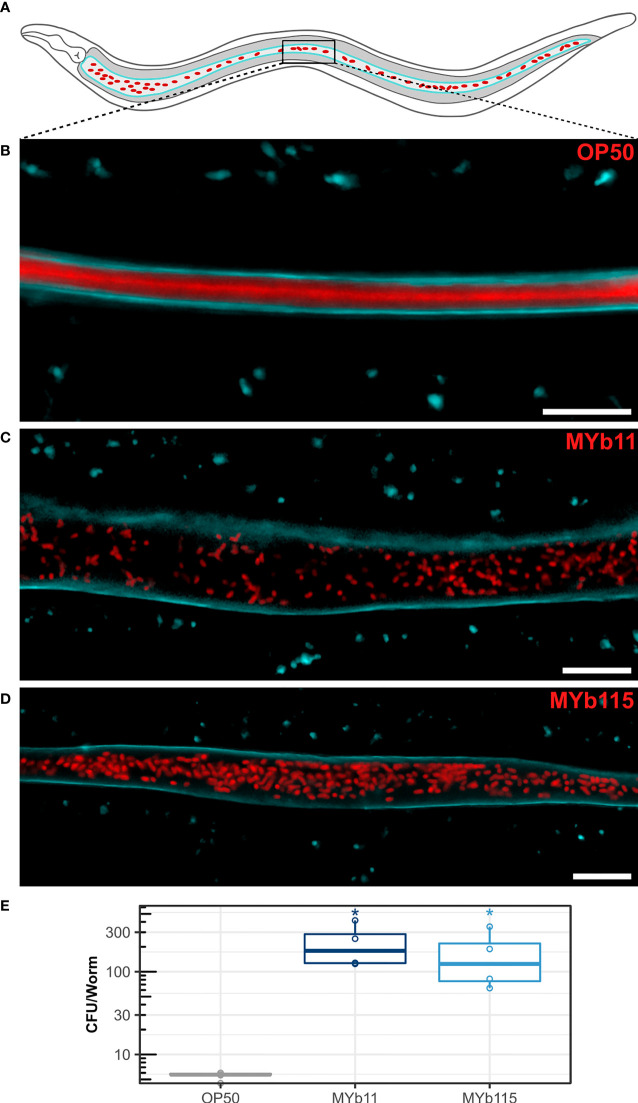
Colonization of the *C. elegans* intestine by MYb11 and MYb115. **(A)** Schematic overview of *C. elegans* intestinal cells (grey) expressing CFP-tagged IFB-2 (cyan) at the apical domain and accumulation of dTomato-tagged bacteria (red) in the intestinal lumen. The box indicates the central area of the intestine where the confocal laser scanning micrographs shown in **(B–D)** were created. All worms used for microscopy **(B–D)** and CFU **(E)** assays were young adult worms (24h post L4 stage). **(B–D)** Confocal laser scanning micrographs showing optical longitudinal sections through intestinal structures of young adults of the transgenic *C. elegans* strain BJ49 *kcls6* (IFB-2::CFP) that had been exposed to fluorescently tagged **(B)** *E*. *coli* OP50, **(C)** *P. lurida* MYb11, or **(D)** *P. fluorescens* MYb115. The dTomato fluorescence of the bacteria is shown in red, and the CFP fluorescence at the apical domain of the intestine is shown in cyan. Scale bars = 10 µm. **(E)** Bacterial load was measured in colony-forming units (CFUs) per worm (n=4 biological replicates), and compared to the CFU of OP50 control *P* values are considered significant and denoted with asterisks according to *p < 0.05, as determined by Wilcoxon-Rank Sum analysis with FDR correction. Also see **Supplementary Figure S2** and **Supplementary Table**.

We next investigated colonization of the *C. elegans* intestine by MYb11 and MYb115. Using confocal laser scanning microscopy and fluorescently labeled MYb11, MYb115, and OP50 strains, we confirmed that MYb11 and MYb115 are able to colonize the intestine of young adult worms (Zimmermann et al., 2019; Dirksen et al., 2020; Pees et al., 2021a) and visualized for the first time the colonization of the worm intestine by MYb11 and MYb115 at single cell resolution (**Figures 2A–D** and **Figure S1**). While we could not observe single, intact bacteria but only diffuse dTomato fluorescence in the intestines of young adult worms that had been exposed to OP50 (**Figure 2B**), nearly all MYb11 and MYb115 bacteria present in the intestines of young adult worms were intact (**Figures 2C, D**, **S1**). Also, visual observations of worms exposed to MYb11 and MYb115 suggest that the intestinal lumina were distended in comparison to the narrow intestinal lumina of worms on OP50. In addition, we quantified the colonization of *C. elegans* by MYb11 and MYb115 by counting colony-forming units (CFUs) (**Figures 2E**, **S2**). Both microbiota isolates, MYb11 and MYb115, showed increased colonization in the intestine at day 1 of adulthood when compared to the control OP50 (**Figures 2E**, **S2**). Furthermore, as a first step towards monitoring microbiota colonization in ageing *C. elegans*, we assessed bacterial load at different days of adult lifespan and observed a gradual increase in the CFU counts as the worm ages (**Figure S2**).

### MYb11 Protects *C. elegans* Against Bt679 Infection but Increases Susceptibility to Bt679 Toxin Exposure

**Figure 3:**
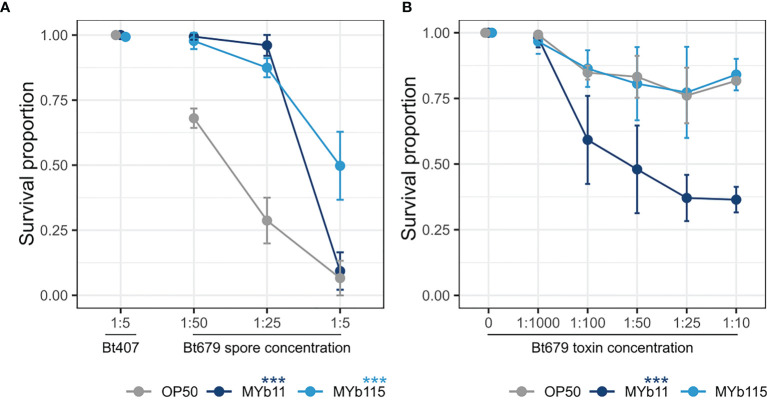
MYb11 and MYb115 protect *C. elegans* from infection with *B. thuringiensis* Bt679, but MYb11 increases susceptibility to purified Bt679 toxins. **(A, B)** Survival proportion of *C. elegans* N2 on different concentrations of *B. thuringiensis* Bt679 **(A)** spore solution and **(B)** purified toxins (containing Cry21Aa3 and Cry14Aa2) mixed with each of the microbiota isolates (MYb11 or MYb115), or OP50 as a control 24 hours post infection. *B. thuringiensis strain* Bt407 (mixed with each of the microbiota isolates independently or OP50) was used as a non-pathogen control in the infection experiments with Bt spores. Data are representative of three independent runs (see **Supplementary Table**). Error bars denote the range of the median of survival proportions of four biological replicates (n = 4), each containing at least 30 worms per treatment condition. Statistical analyses were performed using the GLM framework and Bonferroni correction for multiple testing with the OP50 control treatment group. *P* values are considered significant and denoted with asterisks according to ***p < 0.001.

In parallel to studying *C. elegans* colonization by MYb11 and MYb115 and the associated effects on worm fertility and lifespan, we were also interested in further exploring the protective capacities of MYb11 and MYb115. In particular, we asked whether the previously observed protective effect of MYb11 and MYb115 against Bt247 extends to another Bt strain, MYBt18679 (Bt679) and its toxins. Bt produces crystal pore-forming toxins (Cry PFTs) that are important virulence factors involved in its pathogenesis (Masri et al., 2015), which damage the nematode *via* penetrating the target cell membrane introducing pores and eventually leading to cell death (Zárate-Potes et al., 2020). Different strains of Bt produce distinct Cry PFTs and vary in virulence; Bt247 produces the nematicidal toxin Cry6Ba, whereas Bt679 produces the nematicidal toxins Cry21Aa3 and Cry14Aa2 (Schulte et al., 2010; Masri et al., 2015; Hollensteiner et al., 2017; Papkou et al., 2019; Zárate-Potes et al., 2020). We assessed worm population growth (as a proxy for fitness) and worm survival (as a proxy for resistance) of Bt679 spore-infected N2 worms in the presence of MYb11 and MYb115. We found that worms infected with Bt679 showed an increase in population growth in the presence of both MYb11 and MYb115 (**Figure S3**) and significantly increased survival (**Figure 3A**) compared to infected worms on the *E. coli* OP50 control. These results align with our previous results on the protective effect of these microbiota isolates against infection with Bt247 (Kissoyan et al., 2019). Thus, MYb11 and MYb115 protect not only against Bt247 but also against Bt679. Since specific microbiota isolates can confer protection against pathogens by directly interfering with the function of bacterial toxins [reviewed in Masson and Lemaitre (2017)], we investigated if MYb11 and MYb115 have a direct effect on Bt679 Cry toxins. Thus, we exposed *C. elegans* to a mix of purified Bt679 toxins (containing Cry21Aa3 and Cry14Aa2) in the presence or absence of the microbiota isolates MYb11 and MYb115. Intriguingly, we found that worms on MYb11 showed significantly increased susceptibility to the Bt679 toxins (**Figure 3B**), contrary to the protective effect we observed when infecting worms with Bt679 spores (**Figure 3A**). Worms on MYb115 were as resistant to Bt679 Cry toxins as worms on OP50 (**Figure 3B** and **Supplementary Data File**). However, as worm survival after Bt679 toxin exposure was relatively high for worms on both MYb115 and the OP50 control, it is difficult to draw any conclusion on a potential protective effect of MYb115 against toxin exposure.

## Discussion

We here investigated the effects of the two natural *C. elegans* microbiota isolates, *P. lurida* MYb11 and *P. fluorescens* MYb115, which are protective against pathogen infection, on worm fertility and lifespan. Interestingly, both microbiota isolates affected the reproductive timing and increased early reproduction, while only MYb11 increased overall fertility and decreased lifespan (**Figure 1**). It is known that bacterial-derived metabolites and vitamins can have an effect on *C. elegans* life-history traits. For example, vitamin B12 affects *C. elegans* development and fertility (MacNeil et al., 2013; Watson et al., 2014). Interestingly, both MYb11 and MYb115 are capable of producing vitamin B12 (Zimmermann et al., 2019). It is thus tempting to speculate that these *Pseudomonas* isolates affect reproduction in a similar way. At this point, we can also only speculate about the reason for the negative effect of MYb11 on lifespan (**Figure 1B**). One possible explanation for the decrease in lifespan on MYb11 may be an increase in bacterial colonization and infection in aging worms, as shown previously for the laboratory food bacterium *E. coli* OP50 (Garigan et al., 2002; Portal-Celhay et al., 2012; Podshivalova et al., 2017). Similarly, colonization by MYb11 and MYb115 seems to increase during aging (**Figure S2**). However, while the bacterial load is increased in worms on both MYb11 and MYb115, only MYb11 had a negative effect on lifespan. One possible differential effect of MYb11 and MYb115 on overall fertility and lifespan is the differential production of metabolites from these distinct bacterial species (Zimmermann et al., 2019). Which microbiota-derived metabolites affect *C. elegans* fertility and lifespan needs to be determined in the future.

Our interest in the two microbiota isolates, MYb11 and MYb115, originates from our interest in understanding mechanisms of microbiota-mediated protection from pathogen infection. We have previously demonstrated that MYb11 and MYb115 efficiently protect *C. elegans* from infection with *B. thuringiensis* and *P. aeruginosa* (Kissoyan et al., 2019). MYb11 protects the worm against Bt-spore infection by producing the antimicrobial compound massetolide E that inhibits the growth of Bt. MYb115 does not directly inhibit pathogen growth, and the mechanism of MYb115-mediated protection is still unclear. Certain bacteria protect the host against infection by inactivation of pathogen-derived toxins. For example, the probiotic *Bacillus clausii* strain O/C produces proteases that inhibit the cytotoxic effects of *Clostridium difficile* and *Bacillus cereus* toxins *in vitro* (Ripert et al., 2016). Here, we thus investigated if the protective effect of MYb11 and MYb115 against Bt spores extends to exposure with purified Bt toxins. Interestingly, MYb115 did not affect worm survival upon Bt toxin exposure, but MYb11 enhanced *C. elegans* susceptibility to the toxins. This result supports the idea that MYb11-mediated protection, which is effective against Bt spores but not against purified toxins, is mainly mediated by direct inhibition of pathogen growth (Kissoyan et al., 2019). However, MYb11-colonized worms are not protected against the purified Bt toxins, but on the contrary, are more susceptible to toxin exposure. One explanation for this observation may be that MYb11 exploits Bt toxin-induced epithelial damage to invade extra-intestinal tissue and thus becomes pathogenic. Alternatively, Bt toxin activity may be increased in the presence of MYb11. In the gypsy moth *Lymantria dispar*, it was shown that Bt killing requires the host resident microbiota member *Enterobacter* sp. in the insect hemolymph (Broderick et al., 2006; Broderick et al., 2009). Similarly, the Bt toxin Cry1Ca drastically alters intestinal microbiota in the lepidopteran host *Spodoptera littoralis*, resulting in the predominance of *Serratia* and *Clostridium* species rendering the resident microbiota pathogenic (Caccia et al., 2016). Also, Caccia and colleagues could demonstrate that midgut bacteria can enter the body cavity through toxin-induced epithelial lesions. Thus, these studies highlight a potential role of the intestinal microbiota in aggravating the effects of Bt toxins. It should be noted that purified Bt679 toxins do not kill worms as efficiently as Bt spores and that worm survival is relatively high (**Figure 3**). The presence of MYb11, however, enabled killing. In contrast, worms on MYb115 survived as well as control worms on *E. coli* OP50. Although it is difficult to draw any conclusion on a protective effect of MYb115 under these conditions, this microbiota isolate seems to have less pathogenic potential than MYb11. It remains to be determined how MYb11 increases *C. elegans* susceptibility to Bt toxins and whether extraintestinal colonization plays a role.

In conclusion, we visualize colonization of *C. elegans* at day 1 of adulthood by microbiota isolates *P. lurida* MYb11 and *P. fluorescens* MYb115, which protect the worm against pathogen infection, at single cell resolution. We show that MYb11 has a positive effect on fertility and a negative effect on lifespan. Moreover, MYb11 decreases *C. elegans* survival when worms are exposed to purified Bt679 toxins. Thus, MYb11 has the potential to become pathogenic to the host in some contexts. Overall, this work highlights that certain *C. elegans* microbiota members can be beneficial and costly to the host in a context-dependent manner.

## Data Availability Statement

The original contributions presented in the study are included in the article/**Supplementary Material**. Further inquiries can be directed to the corresponding author.

## Author Contributions

KD secured funding and supervised the work. KD, KK, and LP conceived the study. KK, LP, CG, and BP designed and performed the experiments and analyzed the data. IH performed experiments. JM performed the confocal laser scanning microscopy visualizations. KD, KK, LP, and HS discussed and interpreted the data and wrote the manuscript.

## Funding

This work was funded by the German Science Foundation DFG (Collaborative Research Center CRC1182 Origin and Function of Metaorganisms, project A1.2 to KD and project A1.1 to HS), Germany. We thank the Caenorhabditis Genetics Center (University of Minnesota, Minneapolis, Minnesota, USA), funded by the NIH Office of Research Infrastructure Programs (P40OD010440).

## Conflict of Interest

The authors declare that the research was conducted in the absence of any commercial or financial relationships that could be construed as a potential conflict of interest.

## Publisher’s Note

All claims expressed in this article are solely those of the authors and do not necessarily represent those of their affiliated organizations, or those of the publisher, the editors and the reviewers. Any product that may be evaluated in this article, or claim that may be made by its manufacturer, is not guaranteed or endorsed by the publisher.
