# Supplementary material for: Exploring Effects of C. elegans Protective Natural Microbiota on Host Physiology
Source: Front Cell Infect Microbiol. 2022 Feb 14;12:775728. doi: 10.3389/fcimb.2022.775728 (PMC8884406; doi:10.3389/fcimb.2022.775728)
Supplement: Supplementary file 1 [file DataSheet_1.pdf]

Supplementary Figures

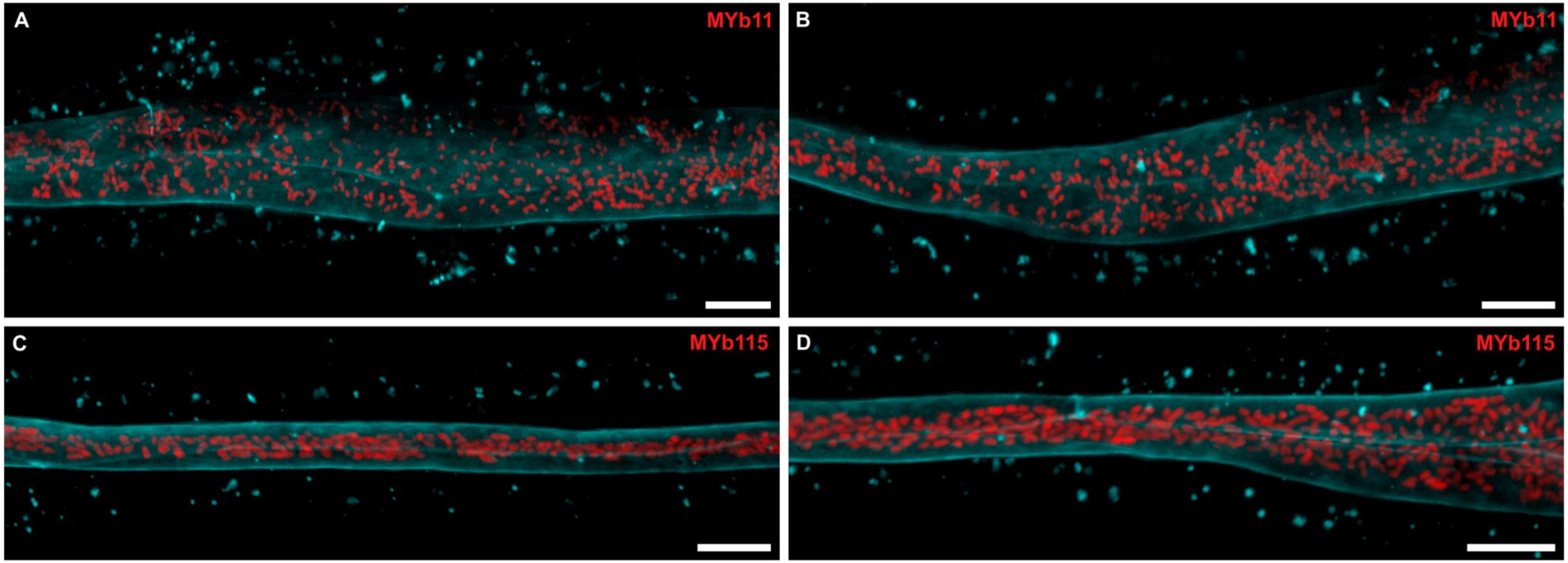

Supplementary Figure S1 Colonization of the *C. elegans* intestine by MYb11 and MYb115. Confocal laser scanning micrographs (maximum intensity projections) showing intestinal structures of young adult worms that had been exposed to **(A, B)** *P. lurida* MYb11 or **(C, D)** *P. fluorescens* MYb115. The dTomato fluorescence of the bacteria is shown in red, and the CFP fluorescence of the intestinal structures is shown in cyan. The intestinal structures were either **(A, C)** in the central or **(B, D)** in the posterior intestine. Scale bars = 10 µm.

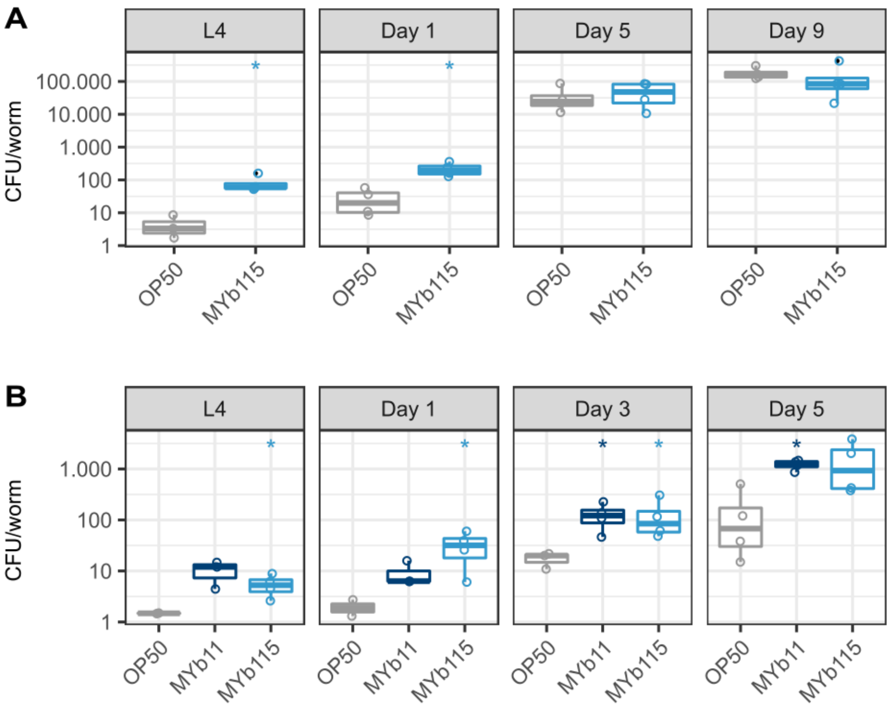

Supplementary Figure S2 Bacterial colonization of *C. elegans* at the L4 stage and different days of adult lifespan grown on MYb11, MYb115, and OP50. Bacterial load was measured at the given time points as colony-forming units (CFUs) per worm. *P* values are considered significant and denoted with asterisks according to *$p < 0.05$, as determined by GLM analysis with Tukey multiple comparison test and FDR correction. Shown are two independent experimental runs (n = 4 technical replicates).

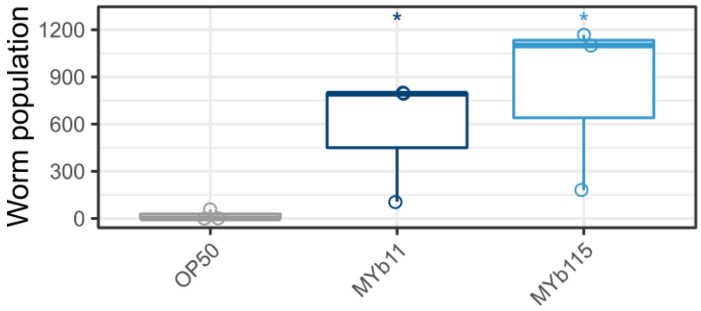

Supplementary Figure S3 MYb11 and MYb115 increase *C. elegans* fitness in the presence of the pathogenic *B. thuringiensis* strain Bt679. The population size of the worms was measured in the presence of pathogenic *B. thuringiensis* Bt679 on each of the protective microbiota isolates *P. lurida* MYb11 and *P. fluorescence* MYb115 and the control food bacterium *E. coli* OP50. Three L4 larvae were picked into the infection plates with lawns containing each treatment bacteria adjusted to an OD_{600nm} of 10 mixed with the Bt679 spores. Worm population size was measured after an incubation period of 5 days at 20°C (n = 3 independent runs). Statistical analysis was performed compared to the OP50 worms, using the Wilcoxon-Rank Sum Test followed by FDR correction for multiple testing **(Supplementary file)**. *P* values are considered significant and denoted with asterisks according to * $p < 0.05$
